# Gradient Anisotropic Natural Rubber-PNIPAM Composite Hydrogels for Programmable NIR-Responsive Actuation

**DOI:** 10.3390/gels12060550

**Published:** 2026-06-19

**Authors:** Qing Zhang, Xueliang Feng, Yuxin Yan, Lin Chen, Honghua Fan, Wenjing Zhou, Kaipeng Li, Xiaohong Yang, Xueyu Du, Chunxin Ma

**Affiliations:** 1 State Key Laboratory of Marine Resource Utilization in South China Sea, School of Chemistry and Chemical Engineering, Hainan University, Haikou 570228, China; 22220856010015@hainanu.edu.cn (Q.Z.); 22220856010033@hainanu.edu.cn (X.F.); 24220856020063@hainanu.edu.cn (Y.Y.); 22110805000014@hainanu.edu.cn (L.C.); fhonghua@126.com (H.F.); zwjing666@163.com (W.Z.); duxueyu@hainanu.edu.cn (X.D.); 2 Key Laboratory of Quality Safe Evaluation and Research of Degradable Material, State Administration for Market Regulation, Hainan Academy of Inspection and Testing, Haikou 570203, China; 13518838751@163.com; 3 Natural Rubber Research & Development Center of Hainan Province for Deep Processing Products, Ledong 572500, China

**Keywords:** smart hydrogel, gradient anisotropy, stimuli-responsive actuation, soft robotics, composite materials

## Abstract

Heterogeneous hydrogels capable of complex, programmable deformation are highly desirable for soft actuators, yet general strategies that simultaneously impart structural anisotropy, rapid responsiveness, and mechanical robustness remain limited. Here, a gradient anisotropic natural rubber-poly(N-isopropylacrylamide) (NR-PNIPAM) composite hydrogel is developed through a simple one-pot polymerization strategy by coupling pH-regulated colloidal stability with gravity-directed redistribution of natural rubber latex particles. Under an optimized pH window, NR nanoparticles gradually migrate during gelation and are fixed as a continuous gradient within the PNIPAM network, generating built-in structural asymmetry for nonuniform deformation. Meanwhile, NR nanoparticles act as soft reinforcing domains to improve mechanical strength, while water-soluble graphene nanosheets provide efficient photothermal conversion for remotely-controlled near-infrared (NIR)-responsive actuation. Benefiting from this synergistic design, the hydrogel exhibits programmable bending and localized folding with high actuation rates of 129° s^−1^ and 46° s^−1^, respectively, along with a tensile strength of 0.32 MPa and an active lifting capability exceeding 70 times its own weight. The material further enables biomimetic gripping and lifting under NIR stimulation. This work establishes a general route to robust gradient hydrogels by integrating colloidal regulation, structural anisotropy, and photothermal actuation, offering a versatile platform for high-performance soft intelligent systems.

## 1. Introduction

Stimuli-responsive hydrogels have emerged as one of the most versatile classes of soft smart materials, owing to their ability to transduce a variety of external signals (e.g., pH, temperature, light, electric fields) into controlled changes in chemical composition and physical geometry [1,2,3]. Among diverse hydrogels, actuating hydrogels can integrate high water content, biocompatibility, and stimuli-responsive shape-changing performance, which can be widely utilized in mini-robotics, artificial muscle devices, biosensors, and controlled drug delivery systems [4,5,6,7]. However, most reported stimuli-responsive actuating hydrogels are based on isotropic polymer networks and therefore mainly undergo simple affine swelling–shrinking deformations [8,9]. This fundamental limitation greatly restricts their utility in applications requiring complex biomimetic motions, such as grasping, crawling, and three-dimensional (3D) shape transformations [10,11]. Inspired by the anisotropic architectures and sophisticated motions of living organisms [12,13], various anisotropic actuating hydrogels have been developed in recent years [14,15,16], enabling increasingly complex deformation modes [17,18,19]. Representative strategies for constructing anisotropic hydrogel structures include photolithographic patterning [20,21], microfluidic spinning [22,23], directional freezing [24,25], directional electrospinning [26,27], and electric-field-induced alignment [28,29]. Although these approaches have led to important advances in anisotropic hydrogel design, they generally rely on complex multistep fabrication procedures or specialized equipment [30,31]. Moreover, many of these methods are not sufficiently universal and are often limited to specific material systems or stimulus-response modes. More importantly, most anisotropic hydrogels still suffer from weak mechanical strength, typically with tensile strengths below 0.1 MPa, which severely limits their actuation force output [32,33]. Therefore, there remains an urgent need for simple and general fabrication strategies that can simultaneously impart anisotropic structures for complex actuation and robust mechanical performance for powerful actuation output [34,35].

Natural rubber (NR) is an attractive candidate for addressing this challenge because it is a renewable elastomer with excellent mechanical properties and inherent biocompatibility [36,37]. More importantly, natural rubber latex, which contains nanoscale NR particles, can be directly incorporated into hydrogel precursor solutions through simple mixing, thereby providing an efficient route to improve hydrogel mechanical performance. NR latex particles possess a characteristic core–shell structure, consisting of a hydrophobic polyisoprene core and a mixed protein–phospholipid interfacial layer [38]. In alkaline aqueous media, these particles can remain colloidally stable because of electrostatic repulsion arising from negatively charged surface groups associated mainly with proteins and other non-rubber components, including carboxylate (-COO^−^) and amino-related functionalities [39]. As a result, NR nanoparticles can be readily dispersed in aqueous precursor solutions, and their colloidal stability can be effectively regulated by adjusting pH [40,41]. When combined with thermo-responsive polymers such as poly(N-isopropylacrylamide) (PNIPAM), NR nanoparticles can markedly enhance the mechanical properties of actuating hydrogels [42,43]. In particular, NR nanoparticles possess high strength yet relatively low modulus, and compared with rigid inorganic nanofillers, they are more effective at dissipating stress and reducing stress concentration under deformation while preserving network deformability [36,44].

In principle, NR latex also offers a unique opportunity to construct gradient anisotropic hydrogels capable of both complex and powerful actuation, although most previously reported NR-containing actuating hydrogels remain isotropic. On the one hand, NR nanoparticles have a density of approximately 0.92 g cm^−3^, which is lower than that of water. On the other hand, their NR stability in hydrogel precursor solutions can be finely tuned by adjusting pH and precisely evaluated by means of zeta potential measurements at room temperature (the larger the absolute value of the zeta potential, the stronger the electrostatic repulsion between the NR-nanoparticles and the more stable the NR latex) [39]. In addition, the Zeta potential measurements of the natural rubber dispersion were performed only at room temperature, as it can show negligible temperature dependence at the range of 0–50°C [40,45]. Therefore, under appropriate pH conditions, the NR nanoparticles can achieve intermediate stability during polymerization: they are not so unstable that they undergo rapid upward sedimentation or aggregation, yet they are not so stable that they remain uniformly dispersed throughout the matrix. Instead, they can gradually redistribute along the gravity direction and form a continuous concentration gradient from the lower region to the upper region of the hydrogel. Such a gradient distribution, similar to uniform dispersion, can still effectively alleviate stress concentration in the hydrogel network and substantially improve mechanical properties [46,47]. More importantly, compared with conventional bilayer hydrogels, this continuous gradient architecture is expected to exhibit much better structural stability by avoiding interlayer delamination during repeated large-amplitude stimuli-responsive deformation cycles [48,49]. Furthermore, this method is universal and can be applied to various other charged nanoparticle dispersions (e.g., synthetic rubber latex, nanoclay dispersion, etc.) for the preparation of gradient-structured hydrogels.

In this work, we develop a simple and general strategy for fabricating an anisotropic NR-PNIPAM composite hydrogel with a continuous gradient structure. We directly disperse NR latex into the N-isopropylacrylamide precursor solution and adjust the pH at room temperature to obtain an appropriate value for the colloidal stability of NR nanoparticles, which can be precisely evaluated via zeta potential measurements, enabling one-step free-radical polymerization to fix a gradient distribution of NR nanoparticles along the gravity direction. The resulting gradient NR-PNIPAM hydrogel integrates complex programmable actuation with robust mechanical performance. First, the anisotropic gradient structure enables programmable deformation modes, including bending and localized folding. Second, different from the gradient distribution of rigid inorganic nanoparticles/nanosheets, that of these relatively soft and tough NR nanoparticles can effectively disperse external stress and reduce stress concentration in the hydrogel network to significantly enhance its mechanical properties, including tensile strength and elongation at break, while maintaining excellent structural stability under repeated large deformations. Therefore, unlike existing robust but isotropic NR-composite hydrogels, gradient but low-strength hydrogels, and layered hydrogels prone to interlayer delamination, this NR-composite hydrogel can integrate a gradient anisotropic structure, highly enhanced mechanical strength, and excellent interlayer stability. In addition, the incorporation of water-soluble graphene nanosheets with high photothermal conversion efficiency from the visible to the near-infrared (NIR) spectral range can endow the hydrogel with rapid non-contact light responsiveness [35,50]. As a result, this NR-PNIPAM hydrogel can simultaneously achieve excellent photothermal-responsive biomimetic behaviors, with not only programmable complex bending and folding actuation but also relatively powerful actuating force owing to its highly enhanced mechanical performance.

## 2. Results and Discussion

### 2.1. Fabrication Strategy and Structural Design of Gradient NR-PNIPAM Composite Hydrogels

**Figure 1:**
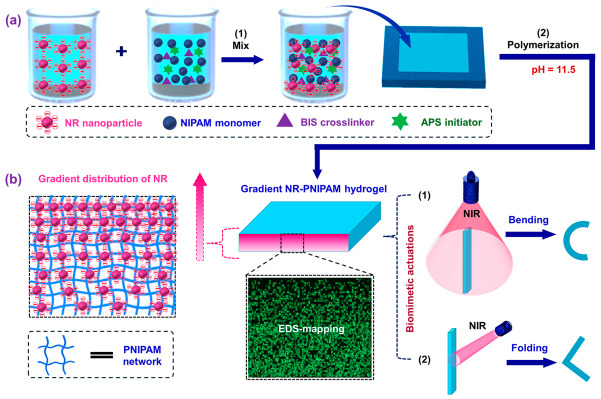
Schematic illustration of the preparation, gradient structure, and NIR-responsive actuation of the NR-PNIPAM composite hydrogel. (**a**) Natural rubber latex was mixed with the PNIPAM precursor solution and cast into a flat mold, followed by in situ polymerization at pH 11.5 to form the composite hydrogel. (**b**) The resulting hydrogel exhibits a gradient distribution of NR nanoparticles in the PNIPAM network, generating an asymmetric structure verified via EDS mapping, which enables biomimetic bending and localized folding under NIR irradiation.

To realize a hydrogel actuator that simultaneously combines structural anisotropy, robust mechanical performance, and rapid photothermal responsiveness, we designed a natural rubber-poly(N-isopropylacrylamide) (NR-PNIPAM) composite hydrogel through a one-pot in situ polymerization strategy. As illustrated in Figure 1a, natural rubber latex was directly mixed with the PNIPAM precursor solution, and the precursor was subsequently polymerized in a horizontally leveled mold at pH 11.5. Under this condition, the colloidal stability of NR nanoparticles was finely balanced, allowing gradual gravity-directed redistribution during gelation while avoiding severe aggregation. As a result, a continuous gradient structure was formed and fixed within the hydrogel network. This asymmetric distribution was further verified via elemental mapping, and the obtained hydrogel exhibited distinct directional deformation behaviors under NIR irradiation, including global bending and localized folding (Figure 1b). Such a fabrication route is notably simple because it does not require multilayer assembly, external alignment fields, or post-patterning processes, yet it effectively introduces anisotropy into the hydrogel matrix.

### 2.2. Characterization of NR Nanoparticles and Gradient Microstructure of the Composite Hydrogel

**Figure 2:**
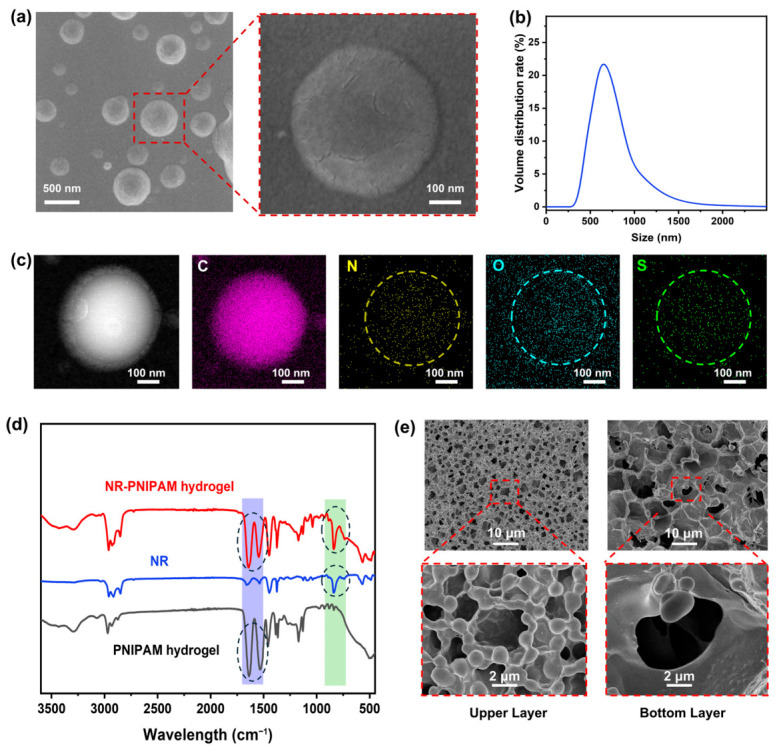
Characterization of NR nanoparticles and the gradient microstructure of the NR-PNIPAM composite hydrogel. (**a**) TEM images of NR nanoparticles at different magnifications. (**b**) Size distribution of the NR nanoparticles. (**c**) HAADF-STEM image and corresponding elemental mappings of C, N, O, and S, revealing the elemental distribution within a representative NR nanoparticle. (**d**) FTIR spectra of the PNIPAM hydrogel, NR, and NR-PNIPAM composite hydrogel. (**e**) Cross-sectional SEM images of the upper and bottom layers of the composite hydrogel, indicating their distinct porous morphologies and structural asymmetry.

The morphology and chemical characteristics of NR nanoparticles, together with the microstructure of the composite hydrogel, were further investigated. TEM observations show that the NR latex consists of well-defined nanoscale particles with relatively uniform morphology (Figure 2a), and the size distribution confirms that these particles are narrowly distributed within the nanometer range (Figure 2b). HAADF-STEM imaging and the corresponding elemental mappings of C, N, O, and S further reveal the compositional heterogeneity of a representative NR nanoparticle (Figure 2c). The strong carbon signal is consistent with a polyisoprene-rich core, while the relatively weak but detectable N, O, and S signals indicate the presence of non-rubber components enriched at the particle interface. Such an elemental distribution is in good agreement with the characteristic core–shell structure of natural rubber latex particles, consisting of a hydrophobic rubber core and a surface layer containing protein- and phospholipid-derived species. In addition, the corresponding EDS spectrum (Figure S1) further confirms the coexistence of C, O, and S, providing complementary evidence for the intrinsic chemical heterogeneity of the NR nanoparticles. FTIR spectra verify the coexistence of NR and PNIPAM in the composite hydrogel (Figure 2d). The characteristic peak of NR at around 835 cm^−1^, attributed to the out-of-plane bending vibration of =C-H in trisubstituted double bonds, confirms the presence of the *cis*-1,4-polyisoprene structure. In addition, the typical absorption bands of PNIPAM, including the amide I band at 1647–1657 cm^−1^ from C=O stretching and the amide II band at 1544–1549 cm^−1^ from coupled N-H bending and C-N stretching, are also clearly observed. The simultaneous presence of these peaks in the NR-PNIPAM spectrum confirms the successful integration of both components into the composite network. More importantly, cross-sectional SEM images reveal obvious differences between the upper and bottom regions of the hydrogel (Figure 2e). The two sides exhibit distinct porous morphologies, confirming that the internal structure is spatially asymmetric rather than homogeneous. This result is consistent with the formation of a gradient microstructure during polymerization and provides an important structural basis for the anisotropic actuation behavior observed later.

### 2.3. pH-Regulated Gradient Formation and Mechanical Performance

**Figure 3:**
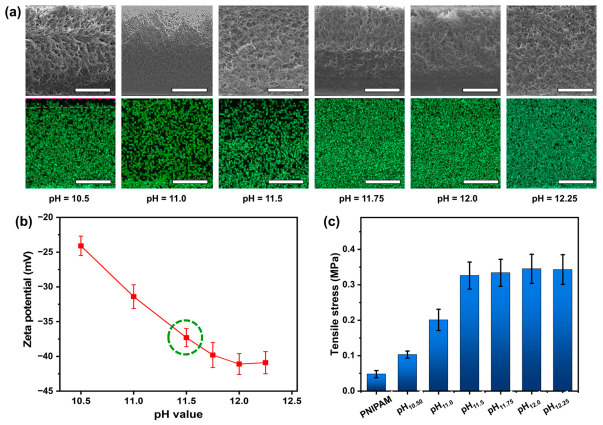
pH-dependent regulation of the gradient structure and tensile properties of NR-PNIPAM composite hydrogels. (**a**) Cross-sectional SEM images and corresponding S elemental mappings of hydrogels prepared at different pH values (from 10.5 to 12.25), revealing the evolution of the asymmetric gradient structure with increasing pH. Scale bars: 200 µm. (**b**) Variation in the zeta potential of NR nanoparticles with pH. (**c**) Tensile stress of the hydrogels prepared at different pH values, showing the influence of pH on the mechanical performance of the composite hydrogels.

Since the formation of the gradient structure is closely related to the colloidal stability of NR nanoparticles, we next examined the effect of pH on particle redistribution and microstructural evolution. Both the cross-sectional SEM images and the corresponding sulfur elemental mappings demonstrate that the hydrogel structure strongly depends on the precursor pH (Figure 3a). More detailed SEM observations of the upper layer, cross section, and bottom layer, together with the corresponding S elemental mappings, are provided in Figure S2, further revealing the pH-dependent evolution of the internal gradient structure. At relatively low pH, the system is insufficiently stabilized, and the sulfur distribution appears less organized, while the microstructure of the upper and bottom layers remains less distinctly differentiated, suggesting partial particle aggregation or limited controllable migration. With increasing pH, the contrast in both morphology and sulfur distribution between different regions of the hydrogel becomes more pronounced, indicating the gradual development of structural asymmetry and more orderly redistribution of NR nanoparticles during gelation. In particular, the cross-sectional S mapping more clearly reveals the emergence of a through-thickness gradient under optimized alkaline conditions. However, when the pH becomes excessively high, the sulfur signal becomes more homogeneous across the sample and the structural asymmetry is weakened, indicating that excessive colloidal stabilization suppresses effective redistribution of NR nanoparticles. This interpretation is supported by the zeta-potential measurements (Figure 3b), where the absolute value of the zeta potential increases with pH, indicating improved colloidal stability of NR nanoparticles in alkaline media. Therefore, pH regulates the competition between mobility, stability, and fixation during polymerization, ultimately determining whether a continuous gradient structure can be formed.

The change in microstructure induced by pH also has a pronounced influence on tensile behavior. As shown in Figure 3c, the tensile stress of the composite hydrogels varies substantially with pH, suggesting that mechanical performance is highly sensitive to the particle distribution state and interfacial interactions within the network. Representative stress–strain curves together with the corresponding Young’s modulus and elongation at break are provided in Figure S3, further illustrating the pH-dependent mechanical regulation induced by NR redistribution. An optimized pH condition yields the best overall tensile response, which can be attributed to the combined effects of effective NR reinforcement and a well-developed gradient architecture. In this state, NR nanoparticles can function as soft reinforcing domains that enhance stress transfer and dissipate energy under deformation, while the continuous gradient structure avoids the weak interfacial boundaries commonly encountered in bilayer or multilayer systems. By contrast, when the pH is too low or too high, the reinforcement efficiency and structural asymmetry are compromised, leading to inferior mechanical properties. These results indicate that pH 11.5 represents a suitable processing window in which colloidal stability and gravity-directed migration are appropriately balanced, enabling simultaneous optimization of anisotropy and toughness. Consistent with this structural regulation, the thermally induced deformation behavior of the hydrogels is also strongly dependent on pH; samples prepared under optimized alkaline conditions exhibit much more pronounced directional bending than those prepared at excessively high pH (Figure S4).

### 2.4. Asymmetric Surface Wettability and NIR Photothermal Properties

**Figure 4:**
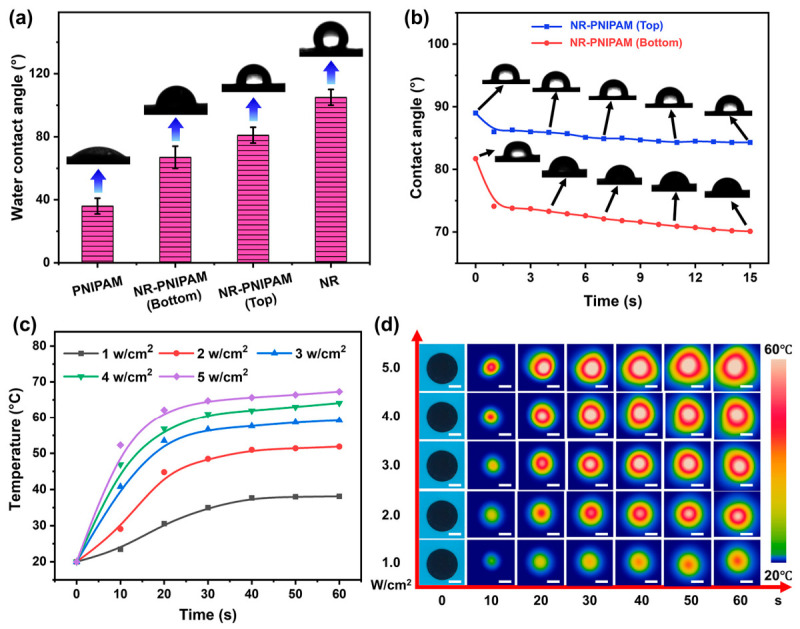
Asymmetric surface wettability and NIR photothermal response of the gradient NR-PNIPAM composite hydrogel. (**a**) Comparison of water contact angles for the PNIPAM hydrogel, the bottom and the upper layer of NR-PNIPAM, and NR, with corresponding water contact angle images shown as insets. (**b**) Dynamic water contact angle changes on the upper and bottom layers of the gradient hydrogel, revealing their distinct wetting behaviors. (**c**) Temperature rise profiles of the composite hydrogel under 808 nm NIR irradiation at various power densities. (**d**) Corresponding infrared thermal images of the hydrogel recorded at different irradiation times and power densities.

In addition to structural asymmetry, the gradient distribution of NR nanoparticles also induces distinct surface properties on the two sides of the hydrogel. As shown in Figure 4a, the water contact angles of pristine PNIPAM hydrogel, NR, and the upper and bottom surfaces of the NR-PNIPAM composite are clearly different. The composite hydrogel exhibits asymmetric surface wettability, with the two opposite surfaces showing distinguishable contact-angle values. This difference reflects the uneven spatial distribution of NR-rich and PNIPAM-rich components across the sample thickness. The dynamic wetting behavior further confirms this asymmetry. The water contact angles on the upper and bottom layers evolve differently with time, suggesting distinct interfacial compositions and water interaction characteristics for the two surfaces (Figure 4b). Such asymmetric wettability is consistent with the gradient microstructure observed in SEM and elemental mapping, and it further supports the existence of a compositional gradient across the hydrogel thickness. The NIR photothermal performance of the gradient NR-PNIPAM composite hydrogel was subsequently evaluated because efficient light-to-heat conversion is essential for remote actuation. Upon 808 nm laser irradiation, the hydrogel exhibits a rapid and power-dependent temperature rise (Figure 4c). The equilibrium temperature increases with increasing irradiation power density, indicating efficient photothermal conversion of the incorporated water-soluble graphene nanosheets. Infrared thermal images recorded at different irradiation times and power densities (Figure 4d) further demonstrate that the heat generation is both rapid and spatially controllable. A representative photothermal heating and cooling profile under 808 nm irradiation is further shown in Figure S5, confirming the rapid temperature increase of the composite hydrogel upon NIR exposure. This photothermal response is particularly important for PNIPAM-based actuators because local heating above the lower critical solution temperature induces dehydration and contraction of the PNIPAM network, thereby generating internal strain. In the present gradient system, the asymmetric structure transforms this isotropic volume transition into directional shape deformation. Therefore, the combination of graphene-enabled photothermal conversion and NR-induced structural asymmetry provides the physical basis for programmable NIR-triggered actuation.

### 2.5. Programmable NIR-Responsive Actuation and Bioinspired Gripping Performance

**Figure 5:**
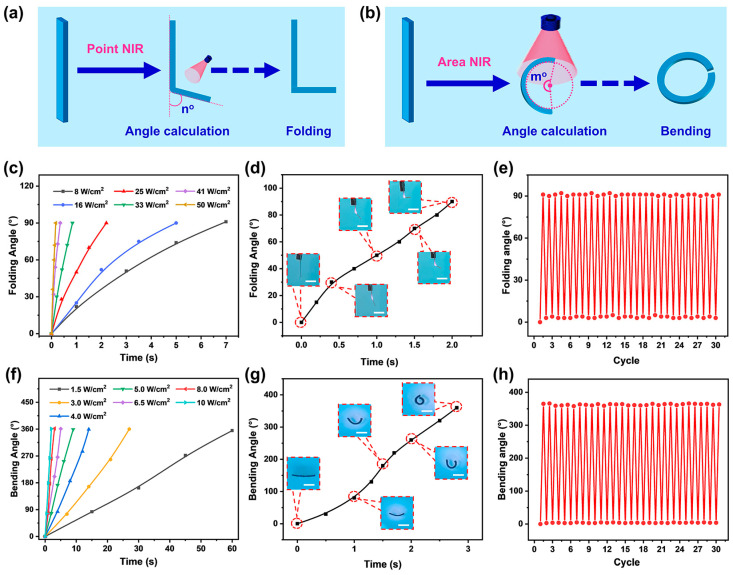
NIR-responsive programmable actuation behavior of the gradient NR-PNIPAM composite hydrogel. (**a**,**b**) Schematic illustrations of localized folding under point NIR irradiation and global bending under area NIR irradiation, together with the corresponding angle definitions. (**c**,**d**) Folding angle as a function of irradiation time at different power densities and representative sequential images of the folding process. (**e**) Reversible folding performance over repeated irradiation cycles. (**f**,**g**) Bending angle as a function of irradiation time at different power densities and representative sequential images of the bending process. (**h**) Reversible bending performance over repeated irradiation cycles.

Based on this structural design, the gradient NR-PNIPAM hydrogel exhibits two distinct yet complementary NIR-triggered actuation modes, namely, localized folding under point irradiation and global bending under area irradiation (Figure 5a,b). In the folding mode, the deformation is highly power-dependent and proceeds within a sub-second to second timescale at relatively high irradiation densities. As shown in Figure 5c, the folding angle increases monotonically with irradiation time and reaches approximately 90° under all tested conditions, while the response time decreases dramatically with increasing power density. Specifically, the hydrogel requires about 7 s to reach a folding angle of 90° at 8 W cm^−2^, whereas this time is shortened to about 5 s at 16 W cm^−2^ and about 2 s at 25 W cm^−2^. Further increasing the power density to 33–50 W cm^−2^ compresses the actuation time to well below 1 s, corresponding to a maximum folding rate of up to 46° s^−1^. Representative time-lapse images (Figure 5d) further confirm a continuous and spatially localized deformation process, where the initially flat strip rapidly bends at the irradiated region and evolves into a sharply folded configuration. Notably, the folding angle remains highly stable during 30 repeated on/off cycles, reversibly switching between nearly 0° and ~90° without obvious amplitude decay (Figure 5e). Such excellent cyclic fidelity indicates that the local stress generated during repeated folding can be effectively accommodated by the NR-reinforced network, thereby suppressing irreversible damage or structural fatigue.

A similar but more amplified tendency is observed in the bending mode under area irradiation. As shown in Figure 5f, the bending angle continuously increases with irradiation time and can ultimately reach ~360°, corresponding to a nearly closed-ring deformation. More importantly, the bending kinetics are markedly accelerated as the power density increases from 1.5 to 10 W cm^−2^. At a low power density of 1.5 W cm^−2^, the hydrogel requires about 60 s to approach 360°, whereas the required time decreases to ~27 s at 3.0 W cm^−2^ and ~12 s at 4.0 W cm^−2^. When the power density is further increased to 5.0, 6.5, 8.0, and 10 W cm^−2^, full bending is achieved within approximately 8, 6, 4.5, and 3 s, respectively, yielding a maximum bending rate as high as 129° s^−1^. The sequential photographs in Figure 5g clearly visualize the progressive curvature evolution from a straight strip to a highly curved ring-like shape, highlighting the smooth and continuous nature of the macroscopic deformation. Meanwhile, the cyclic bending test demonstrates nearly invariant switching between ~0° and ~360° over 30 cycles (Figure 5h), confirming the outstanding reversibility and structural stability of the hydrogel during repeated large-amplitude motion. The detailed time-dependent bending and subsequent recovery behaviors are further summarized in Figure S6. Compared with the localized folding mode, the global bending mode exhibits a much larger deformation amplitude and faster angular evolution, suggesting that area irradiation more effectively activates the through-thickness strain mismatch across the entire strip.

The pronounced difference between folding and bending behaviors further reveals the programmable nature of this gradient hydrogel. Mechanistically, NIR irradiation induces rapid photothermal heating of the graphene-containing PNIPAM matrix, leading to dehydration and contraction of the thermo-responsive network. Because the hydrogel possesses a continuous NR gradient across the thickness, together with asymmetric microstructure and surface wettability, the photothermally induced volume change is intrinsically nonuniform rather than affine. This through-thickness shrinkage mismatch generates an internal bending moment, which is the origin of the directional shape transformation. Under point irradiation, the thermal field is spatially confined, so only a local region experiences significant deswelling and contraction, giving rise to site-specific folding. By contrast, under area irradiation, the contraction mismatch is activated over the full length of the strip, resulting in cooperative global bending with a much larger angular output. Therefore, the same material can be switched between localized and global actuation modes simply by changing the irradiation pattern, without altering the composition or geometry of the actuator. Such irradiation-defined motion programmability, combined with fast response, large bending amplitude, and excellent cycling stability, is highly advantageous for soft robotic systems requiring reconfigurable, remotely controlled, and site-selective operations. In addition, the recovery behavior of the deformed hydrogel under different boundary conditions, including one-end-fixed and unconstrained states, is presented in Figure S7.

**Figure 6:**
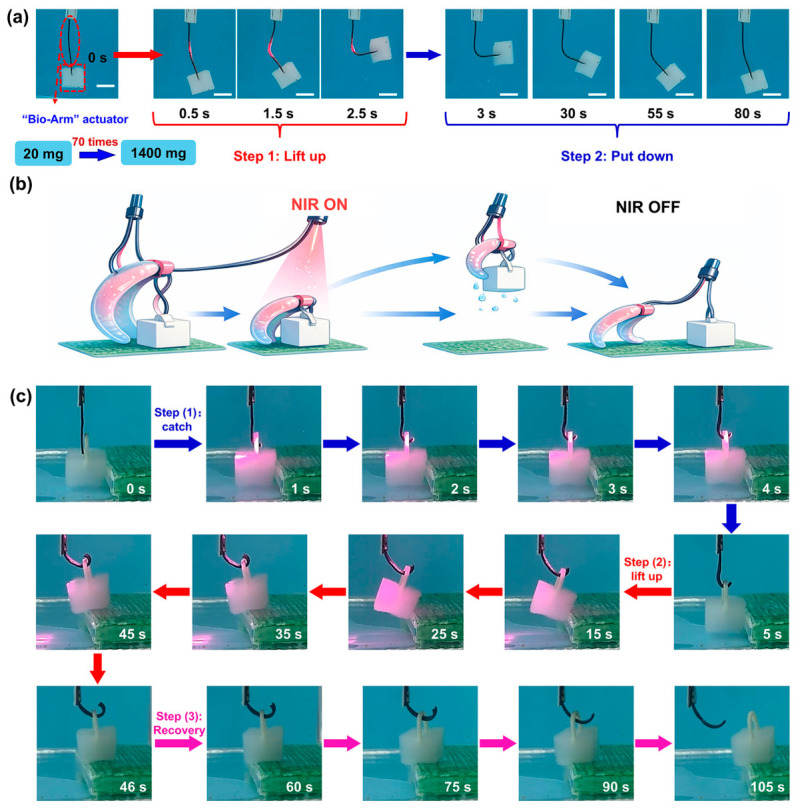
NIR-triggered gripping and lifting performance of the bio-inspired soft actuator based on NR-PNIPAM composite hydrogels. (**a**) Sequential images of the actuator lifting and releasing a load under NIR stimulation. The 20 mg actuator can lift an object weighing up to 1400 mg, corresponding to an active lifting capacity of 70 times its own weight. (**b**) Schematic illustration of the NIR-responsive gripping mechanism. Upon NIR irradiation, the actuator deforms to grasp and lift the target object; after the NIR light is turned off, the actuator relaxes and releases the object. (**c**) Representative time-lapse photographs of the NIR-triggered catching, lifting, and recovery process of the hydrogel actuator. Upon NIR irradiation, the actuator first bends to catch the object, then further deforms to lift it. After the NIR stimulus is removed, the actuator gradually recovers toward its initial shape and releases the object.

To demonstrate the practical utility of the material as a soft actuator, we further constructed a bio-inspired gripping device based on the NR-PNIPAM composite hydrogel. The actuator can grasp, lift, and release an object under NIR stimulation in a fully remote and reversible manner. Notably, a hydrogel actuator with a mass of only 20 mg is capable of lifting a load up to 1400 mg, corresponding to an outstanding active lifting capability of 70 times its own weight (Figure 6a). Under NIR irradiation, photothermal heating induces contraction of the PNIPAM network and drives directional deformation of the anisotropic hydrogel, enabling the actuator to bend toward the target object, catch it, and subsequently lift it (Figure 6b). Once the NIR light is removed, the hydrogel cools, rehydrates, and gradually relaxes back toward its original configuration, thereby releasing the object. The time-lapse images in Figure 6c clearly visualize the full stepwise actuation process, including object catching, lifting, and subsequent recovery/release, confirming the rapid response, reversibility, and operational reliability of the actuator.

The exceptional gripping and lifting performance can be attributed to the synergistic integration of anisotropic architecture and rubber reinforcement. On the one hand, the continuous gradient structure provides the directional deformation required for active grasping. On the other hand, the NR nanoparticles reinforce the hydrogel matrix, improving tensile strength, deformability, and resistance to fracture during repeated large-amplitude motion. Unlike many conventional PNIPAM-based actuators that are limited by low mechanical strength and weak load output, the present NR-PNIPAM system converts photothermal stimulation into both large shape change and substantial mechanical work. To further highlight the strong mechanical output of the actuator, an additional demonstration is provided in Figure S8, where the hydrogel actuator is able to lift a 50 g load, based on the effective mass of the hydrogel segment directly involved in lifting (ca. 0.03 g). Therefore, this material design effectively addresses the long-standing trade-off between actuation complexity and mechanical robustness in hydrogel actuators. Overall, these results demonstrate that regulating the colloidal stability of natural rubber latex within a suitable pH window provides a simple yet powerful route for constructing gradient anisotropic hydrogels. The resulting NR-PNIPAM composite hydrogel simultaneously achieves continuous structural asymmetry, asymmetric surface wettability, efficient NIR photothermal responsiveness, programmable deformation modes, and high active lifting capability. More importantly, the fabrication strategy is general and does not rely on complex processing or interfacial assembly. By coupling gravity-induced nanoparticle redistribution with in situ network formation, this work offers a practical framework for developing next-generation soft actuators that combine biomimetic motion with enhanced mechanical performance.

## 3. Conclusions

In summary, we have developed a simple and effective strategy for fabricating anisotropic NR-PNIPAM composite hydrogels with a continuous gradient structure through one-pot in situ polymerization. By tuning the precursor pH to regulate the colloidal stability of NR nanoparticles, gravity-directed particle redistribution could be precisely controlled and fixed during gelation, leading to a structurally asymmetric hydrogel network without requiring multilayer assembly, external alignment, or complicated processing steps. The resulting gradient hydrogel exhibited distinct differences in microstructure and surface wettability across its thickness, together with improved tensile performance arising from the reinforcing effect of NR nanoparticles. After incorporation of water-soluble graphene nanosheets, the composite hydrogel further displayed rapid NIR photothermal responsiveness and programmable actuation behaviors, including localized folding and global bending under different irradiation modes. Owing to the synergistic integration of anisotropic architecture and rubber reinforcement, the hydrogel actuator showed excellent reversibility, durability, and strong mechanical output. A bioinspired soft gripper constructed from this material was able to grasp and lift objects remotely under NIR stimulation, demonstrating promising active lifting capability and operational reliability. This work establishes a general material design principle based on coupling colloidal stability regulation with gravity-induced nanoparticle migration to create gradient anisotropic hydrogels with enhanced mechanical robustness. Overall, the present strategy not only expands the design toolbox for high-performance actuating hydrogels but also provides a promising platform for future applications in soft robotics, artificial muscles, intelligent manipulation, and other biomimetic soft devices.

## 4. Materials and Methods

### 4.1. Materials

Natural rubber latex (NRL, 60 wt%, pH 10.5 ± 0.1) was purchased from Guangdong Hongke Chemical Raw Material Co., Ltd., Zhongshan, China, N-isopropylacrylamide (NIPAM, 98%) and N,N,N′,N′-tetramethylethylenediamine (TEMED, 99%) were obtained from Shanghai Macklin Biochemical Co., Ltd., Shanghai, China, Sodium hydroxide (NaOH, ≥98%) and poly(sodium 4-styrenesulfonate) (PSS) were purchased from Sigma-Aldrich Trading Co., Ltd. (Shanghai, China). N,N′-methylenebisacrylamide (BIS, 98%) and ammonium persulfate (APS, 99%) were obtained from J&K Scientific Ltd., Beijing, China, Water-soluble graphene dispersion (20 mg/mL) was supplied by Suzhou Tanfeng Graphene Technology Co., Ltd., Suzhou, China, Deionized water was used in all experiments.

### 4.2. Preparation of NR-PNIPAM Gradient Composite Hydrogels

We selected surfactant-modified water-soluble graphene not only because of its excellent aqueous dispersibility but also because of its ultrahigh photothermal conversion efficiency, which stems from graphene’s unique electronic structure with full-spectrum absorption and carrier excitation under light irradiation at a broad range from UV to IR wavelength [35,50]. A graphene aqueous dispersion with a concentration of 1.0 mg/mL was prepared by diluting 500 µL of the original graphene dispersion (20 mg/mL) with 9.5 mL of deionized water before use. The NRL was dissolved with deionized water from 60% to 50% before use.

The precursor dispersion of the hydrogel was prepared at room temperature as follows: (1) Dissolved the NIPAM monomer (100 mg) and BIS crosslinker (3 mg) in 500 µL of 1.0 mg/mL water-soluble graphene dispersion until completely dissolved; (2) added NR latex and graphene: mixed the above precursor solution with 50% NRL at a 1:1 volume ratio to obtain uniform dispersion; (3) adjusted the pH: under continuous stirring, added 5% NaOH solution dropwise while monitoring the pH in real time using a precision pH meter and adjusted the pH of the mixture to different target values (11.0 ± 0.05, 11.5 ± 0.05, 11.75 ± 0.05, 12.0 ± 0.05, and 12.25 ± 0.05); (4) measured zeta potential: took an appropriate amount of the pH-adjusted mixture and measured its zeta potential using a zeta potentiometer to evaluate the colloidal stability of the NR latex (a higher absolute zeta potential indicates better stability); (5) confirmed the status: after confirming that the pH and zeta potential met the desired criteria, the homogeneous precursor dispersion was stored at 4 °C for preparation of this hydrogel.

For hydrogel preparation, 500 µL of the precursor graphene dispersion (1 mg/mL) was mixed with 100 mg NIPAM and 3 mg BIS in a centrifuge tube until complete dissolution. The mixture was precooled at 4 °C for 30 min. Then, 250 µL of NRL and 250 µL of NaOH solution with the desired pH were sequentially added, followed by vigorous shaking to obtain a. Subsequently, 50 µL of APS solution and 5 µL of TEMED were added to initiate polymerization. The precursor solution was gently shaken and immediately injected into a sealed glass mold with a 0.5 mm silicone spacer. The filled mold was kept at 4 °C, and gelation occurred within 30 min. The obtained gels were then further cured at room temperature for 8–14 h. After polymerization, the samples were removed from the mold and immersed in deionized water for 24 h, with the water replaced every 4 h, to remove residual monomers and other soluble impurities. The final products were denoted as NR-PNIPAM gradient composite hydrogels.

### 4.3. Photothermal Characterization

The photothermal performance of the composite hydrogels was characterized under irradiation with an 808 nm NIR laser at various power densities. The temperature change of the hydrogel during irradiation was monitored using an infrared thermal imaging camera. Thermal images were recorded to evaluate the temporal and spatial temperature evolution of the samples under NIR exposure.

### 4.4. NIR-Responsive Actuation

NIR-responsive actuation behavior, including both global bending and localized folding, was evaluated using an 808 nm NIR light source. For bending tests, the samples were exposed to area irradiation, whereas for localized folding tests, point irradiation was applied to designated regions of the hydrogel to induce site-specific deformation. The deformation processes were recorded in real time using a digital camera, and the bending or folding angle, actuation rate, response time, and recovery behavior were analyzed from the captured images or videos. In addition, the point light source was moved to different positions on the sample to assess the programmable local actuation capability of the hydrogels.

### 4.5. Characterizations

Optical images were recorded using a smartphone (Xiaomi 15, Xiaomi Corporation, Beijing, China). The chemical structures of the samples were analyzed via Fourier transform infrared spectroscopy (FT-IR, Nicolet Summit X, Thermo Fisher Scientific, Tewksbury, MA, USA). The average diameter of natural rubber latex nanoparticles was determined via dynamic light scattering (DLS, Zetasizer Nano ZS90, Malvern Panalytical Ltd., Malvern, UK). The morphology of natural rubber particles was observed via transmission electron microscopy (TEM, FEI Talos F200S, Thermo Fisher Scientific, Hillsboro, OR, USA). The microstructure of the samples was characterized via scanning electron microscopy (SEM), and the elemental distribution was analyzed via energy-dispersive X-ray spectroscopy (EDS) mapping (S-4800, Hitachi, Tokyo, Japan). Mechanical properties were measured using a universal testing machine (GP-6113A, Suzhou Gaopin Testing Instrument Co., Ltd., Suzhou, China) at a tensile rate of 10 mm min^−1^, and each hydrogel sample was tested five times in parallel. The surface wettability of the hydrogels was evaluated via contact angle measurements using a contact angle meter (XG-CAMB3, Shanghai Xuanzhun Instrument Co., Ltd., Shanghai, China), with three parallel measurements for each sample. The zeta potential of the samples at different pH values was measured at room temperature using a zeta potential analyzer (Nano ZS90, Malvern Panalytical Ltd., Malvern, UK). The optical transmittance of the hydrogels was measured using a UV-Vis spectrophotometer (752N, Shanghai INESA Analytical Instrument Co., Ltd., Shanghai, China) in the wavelength range of 200–1100 nm. The photothermal heating behavior of the hydrogels under NIR irradiation was recorded with a thermal imaging camera (LH-129, Shenzhen Lianhuicheng Technology Co., Ltd., Shenzhen, China). An 808 nm near-infrared laser (VCL-808nm M1-15W, Beijing Honglan Optoelectronics Technology Co., Ltd., Beijing, China) was used as the irradiation source.

## Data Availability

The data in this work are available from the corresponding author upon reasonable request.

## Supplementary Materials

The following supporting information can be downloaded at https://www.mdpi.com/article/10.3390/gels12060550/s1. Figure S1. Free-radical polymerization of NIPAM; Figure S2: EDS spectrum of NR nanoparticles; Figure S3: pH-dependent evolution of the microstructure and sulfur distribution in NR-PNIPAM composite hydrogels; Figure S4: Tensile properties of PNIPAM and NR–PNIPAM composite hydrogels prepared at different pH values; Figure S5: Thermally induced deformation behaviors of NR-PNIPAM composite hydrogels prepared at different pH values; Figure S6. Transmittance of NR-PNIPAM hydrogels with different graphene concentrations at 808 nm. Figure S7: Photothermal heating behavior of the NR-PNIPAM composite hydrogel under NIR irradiation; Figure S8: Time-dependent bending and recovery behaviors of the gradient NR-PNIPAM hydrogel under area NIR irradiation; Figure S9: Recovery behaviors of the deformed hydrogel under different constraints; Figure S10: Photograph demonstrating the high active lifting performance of the NR-PNIPAM hydrogel actuator.
