# Supplementary material for: Gradient Anisotropic Natural Rubber-PNIPAM Composite Hydrogels for Programmable NIR-Responsive Actuation
Source: Gels. 2026 Jun 19;12(6):550. doi: 10.3390/gels12060550 (PMC13298218; doi:10.3390/gels12060550)
Supplement: Supplementary file 1 [file gels-12-00550-s001.zip › gels-4284162-supplementary.pdf]

Supporting Information

Gradient Anisotropic Natural Rubber-PNIPAM Composite Hydrogels for Programmable NIR-Responsive Actuation

Qing Zhang[1, †], Xueliang Feng[1, †], Yuxin Yan[1], Lin Chen[1], Honghua Fan[1], Wenjing Zhou[1], Kaipeng Li[2], Xiaohong Yang[1]*, Xueyu Du[1], and Chunxin Ma[1,3,*]

[1] State Key Laboratory of Marine Resource Utilization in South China Sea, School of Chemistry and Chemical Engineering, Hainan University, Haikou 570228, China.

[2] Key Laboratory of Quality Safe Evaluation and Research of Degradable Material, State Administration for Market Regulation, Hainan Academy of Inspection and Testing, Haikou 570203, China.

[3] Natural Rubber Research & Development Center of Hainan Province for Deep Processing Products, Ledong 572500, China.

***** Correspondence: (C.M.) machunxin@hainanu.edu.cn; (X.Y.) 990408@hainanu.edu.cn.

† These authors contributed equally to this work.

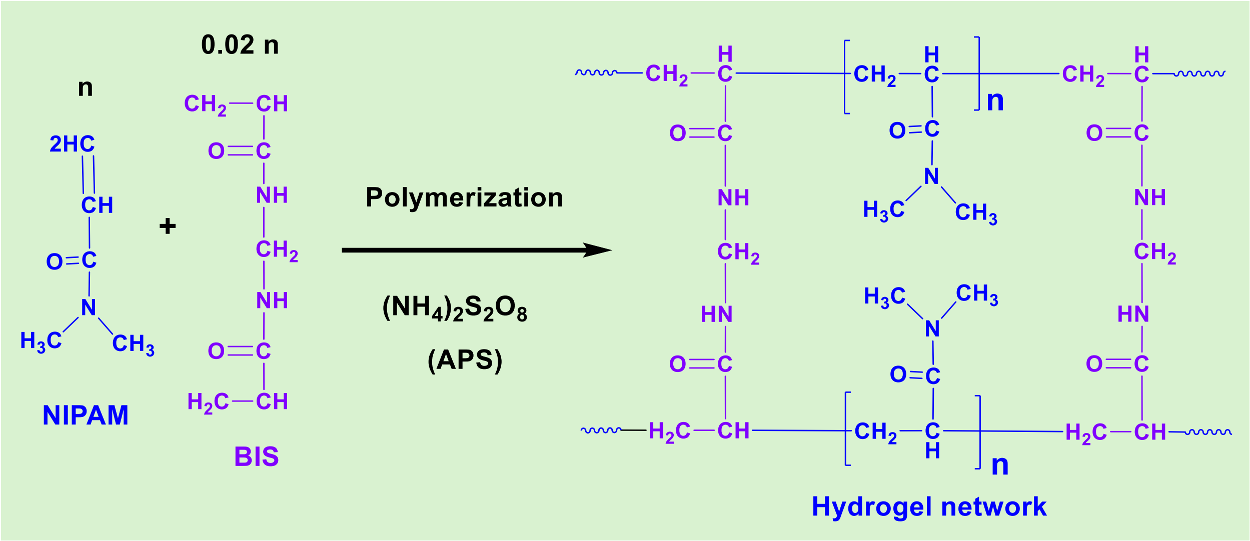

Figure S1. Free-radical polymerization of NIPAM.

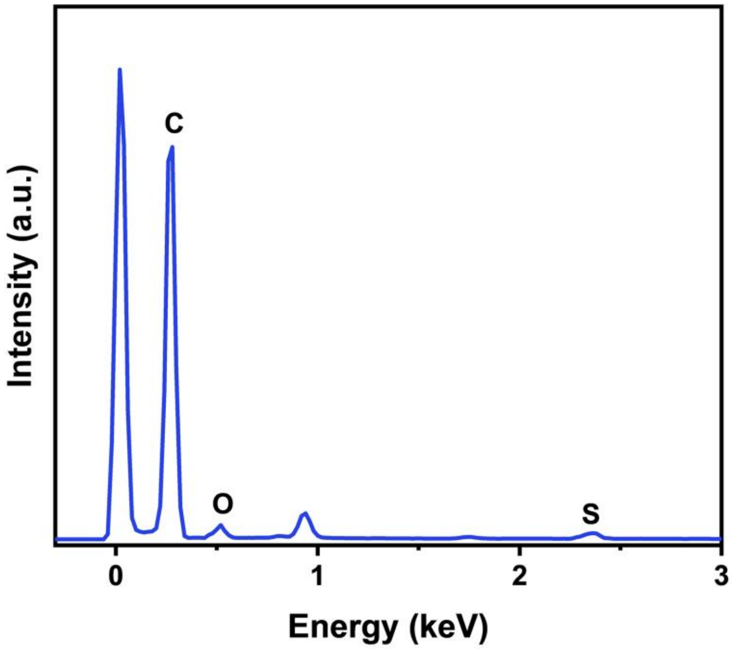

Figure S2. EDS spectrum of NR nanoparticles.

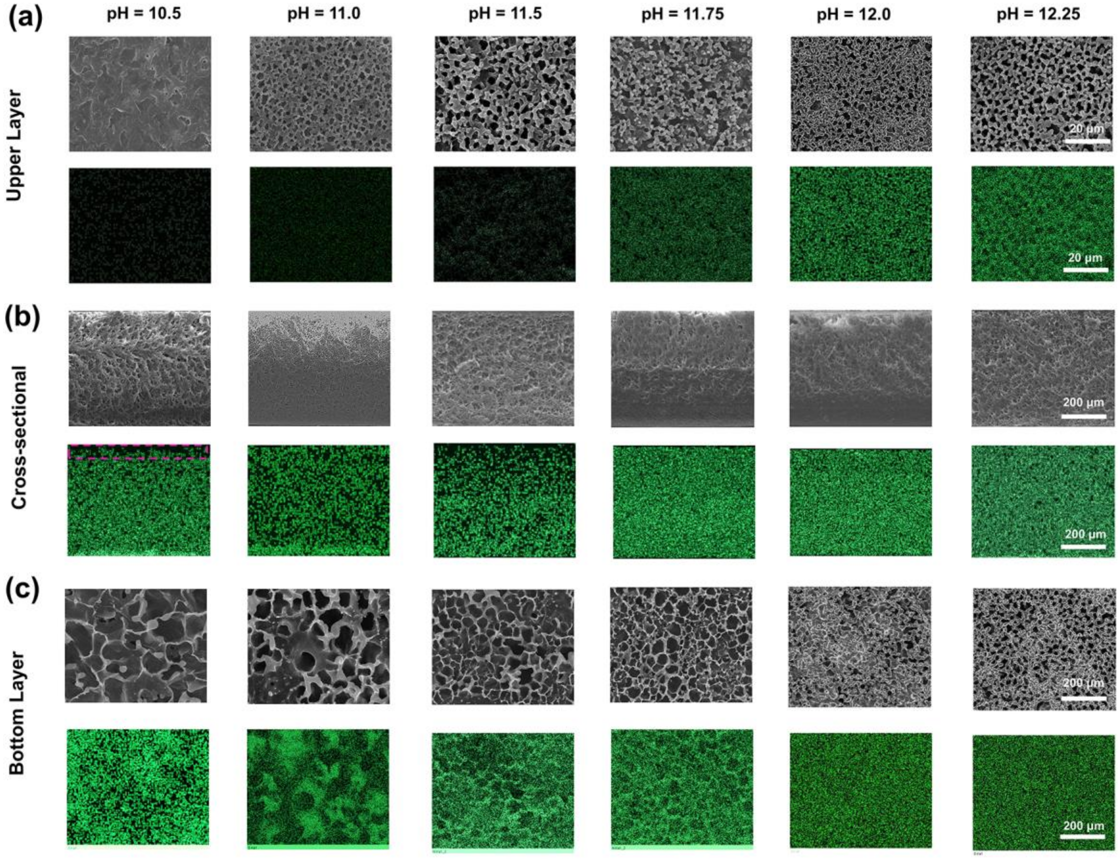

Figure S3. pH-dependent evolution of the microstructure and sulfur distribution in NR-PNIPAM composite hydrogels.

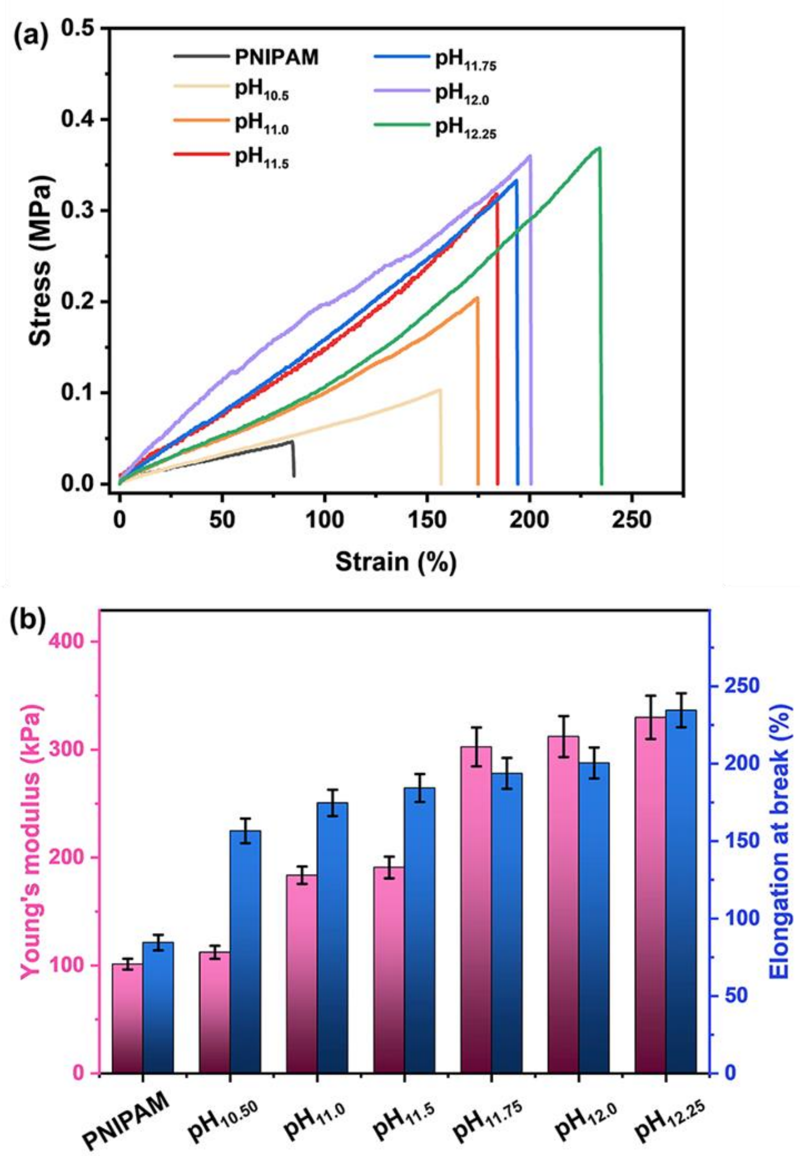

Figure S4. Tensile properties of PNIPAM and NR–PNIPAM composite hydrogels prepared at different pH values.

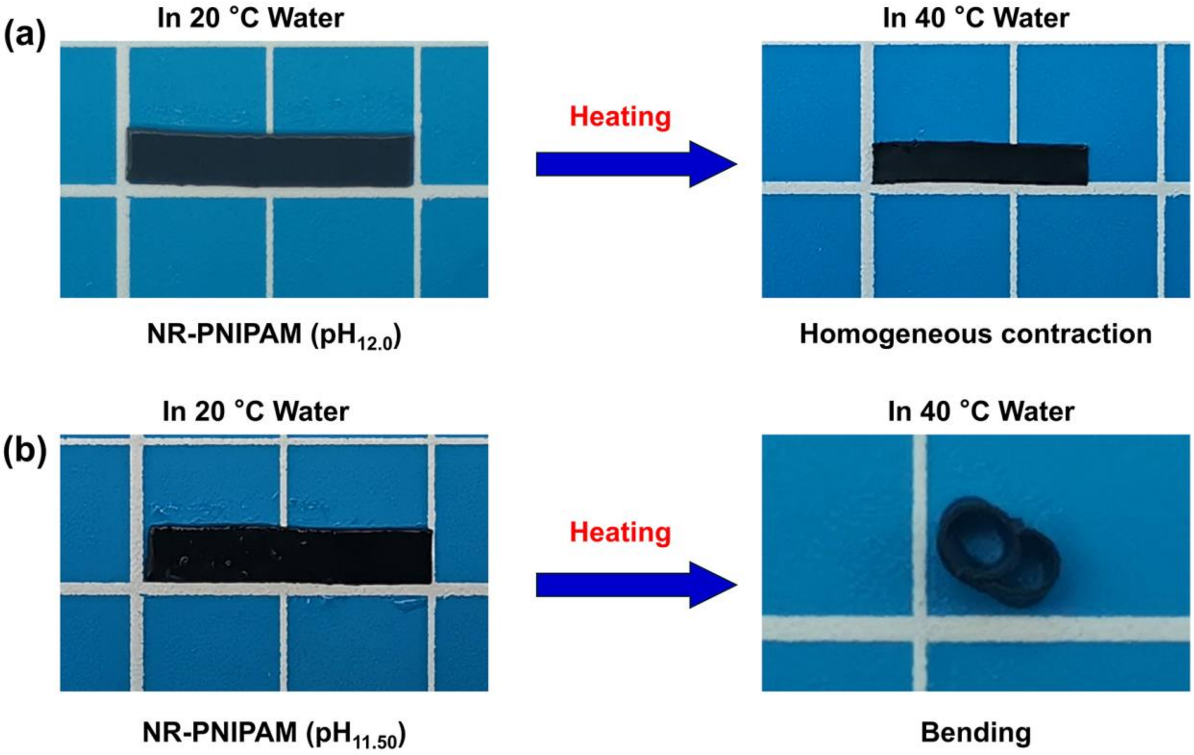

Figure S5. Thermally induced deformation behaviors of NR-PNIPAM composite hydrogels prepared at different pH values.

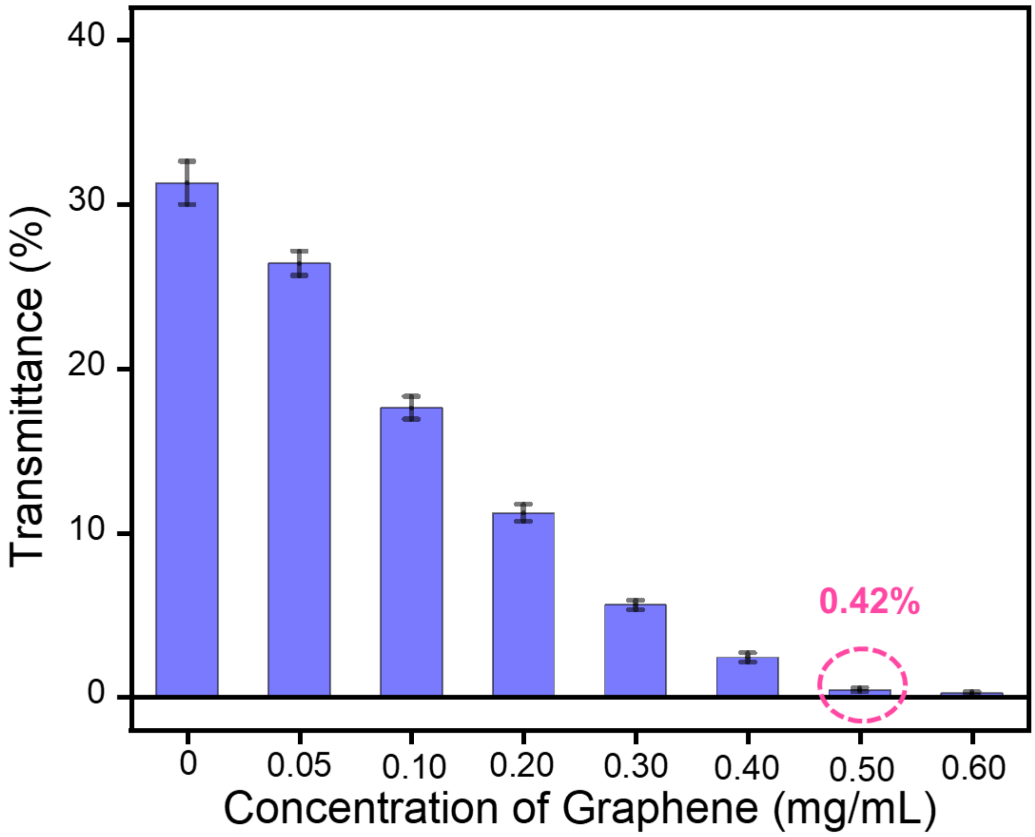

Figure S6. Transmittance of NR-PNIPAM hydrogels with different graphene concentrations at 808 nm.

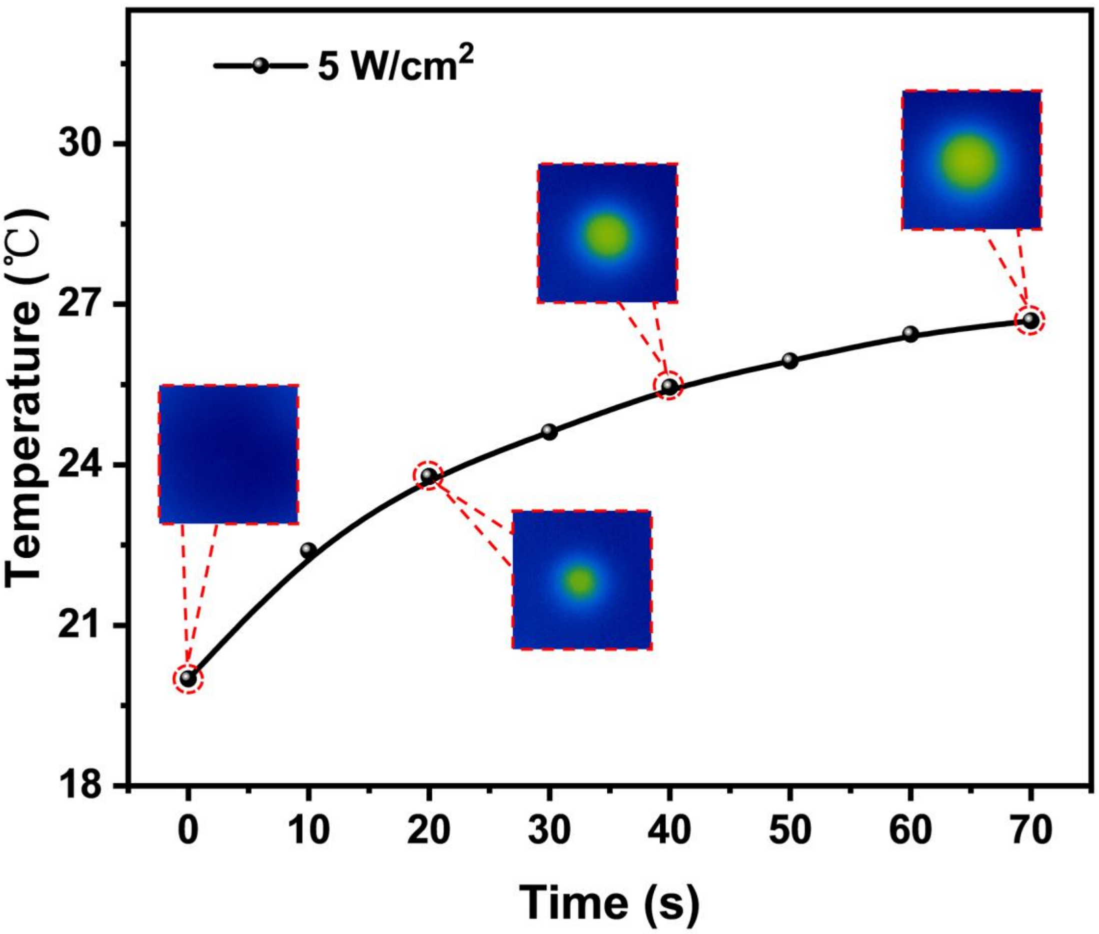

Figure S7. Photothermal heating behavior of the NR-PNIPAM composite hydrogel under NIR irradiation.

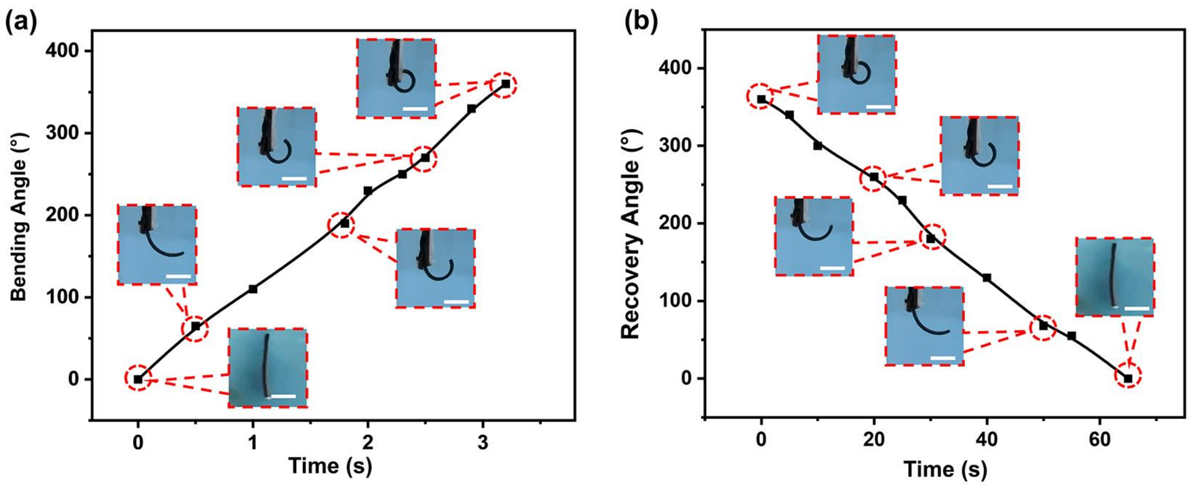

Figure S8. Time-dependent bending and recovery behaviors of the gradient NR-PNIPAM hydrogel under area NIR irradiation.

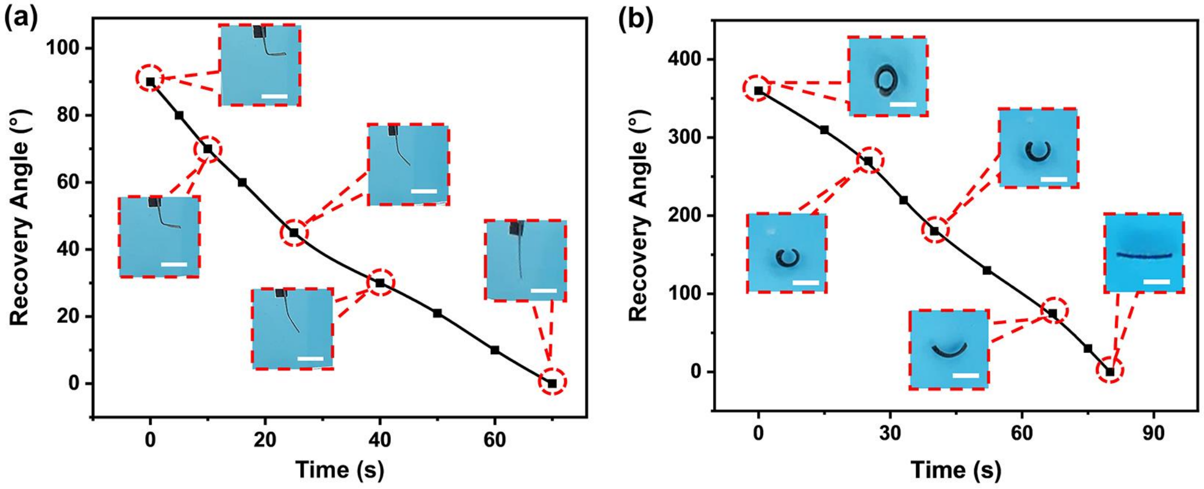

Figure S9. Recovery behaviors of the deformed hydrogel under different constraints.

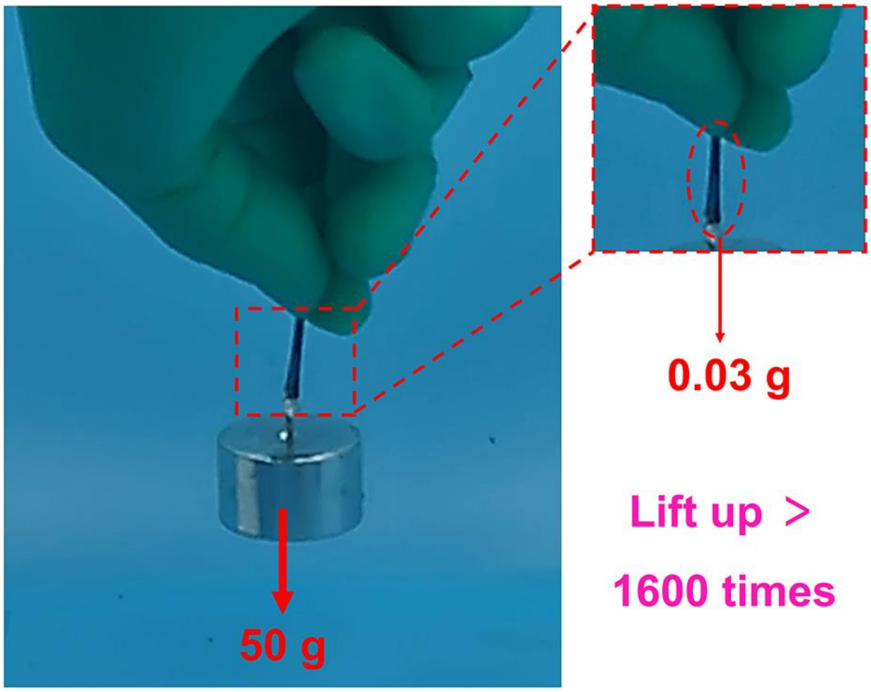

Figure S10. Photograph demonstrating the high active lifting performance of the NR-PNIPAM hydrogel actuator.
